# How peer-review constrains cognition: on the frontline in the knowledge sector

**DOI:** 10.3389/fpsyg.2015.01706

**Published:** 2015-11-03

**Authors:** Stephen J. Cowley

**Affiliations:** Centre for Human Interactivity and the COMAC Cluster–Department of Language and Communication, University of Southern DenmarkSlagelse, Denmark

**Keywords:** peer-review, distributed language, distributed cognition, languaging, ecological psychology, embodied cognition, representation, epistemology

## Abstract

Peer-review is neither reliable, fair, nor a valid basis for predicting ‘impact’: as quality control, peer-review is not fit for purpose. Endorsing the consensus, I offer a reframing: while a normative social process, peer-review also shapes the writing of a scientific paper. In so far as ‘cognition’ describes enabling conditions for flexible behavior, the practices of peer-review thus constrain knowledge-making. To pursue cognitive functions of peer-review, however, manuscripts must be seen as ‘symbolizations’, replicable patterns that use technologically enabled activity. On this bio-cognitive view, peer-review constrains knowledge-making by writers, editors, reviewers. Authors are prompted to recursively re-aggregate symbolizations to present what are deemed acceptable knowledge claims. How, then, can recursive re-embodiment be explored? In illustration, I sketch how the paper’s own content came to be re-aggregated: agonistic review drove reformatting of argument structure, changes in rhetorical ploys and careful choice of wordings. For this reason, the paper’s knowledge-claims can be traced to human activity that occurs in distributed cognitive systems. Peer-review is on the frontline in the knowledge sector in that it delimits what can count as *knowing*. Its systemic nature is therefore crucial to not only discipline-centered ‘real’ science but also its ‘post-academic’ counterparts.

## Introduction

Recent decades have seen shifts in the academy –changes in how people view science, cognition, language and, for related reasons, the nature of social practices. For many, academics no longer strive to unlock the secrets of art and nature but, rather, as professionals in a knowledge sector, they combine teaching with research. A world of what Ziman (2000) calls post-academic science is altering the values of the discipline based research of the last century. For Ziman (2000, p. 173), the interdisciplinary focus of post-academic science is organized by market principles and dedicated to the accomplishment of practical goals. Others report on similar changes: Mirowski (2011) challenges the marketization of science and Readings (1996) decries the emergence of a ‘post historical’ university. These changes in academia are due, in part, to markets, information technology, globalization, and the digitalization of documents (often, bizarrely, referred to as ‘knowledge’)^1^.

In pursuing such changes, like Ziman (2000), I focus on what self-defined communities regard as *scientific* knowledge.^2^ Taking a broad view of knowing, I focus on editorial peer-review. Having overviewed the literature of the field (while striving to be ‘disinterested’), I suggest that peer-review is *more* than normative. Rather, peer-review is *cognitive* in a precise sense; it creates enabling/disabling conditions for the flexible behavior that shapes academic publications. Peer-review uses, not just Merton’s (1942) scientific values, but also how editors, authors, reviewers and others adapt as they enact institutional practices. At coarse levels of granularity, all parties affect knowledge-making. Reviewers are crucial in, at least, Ziman’s (2000) *real* science –value driven practice that contrasts with what he calls the Legend.^3^ By real science, Ziman (2000) focuses on practices whereby research communities define the assumptions and knowledge that is taken to constitute the framework of a scientific discipline.

### Cognition and Knowledge

Historically, individual cognition was opposed to the knowledge that a group hold common. In the West, the dominant view of ‘cognition’ built on rationalist–empiricist debate and, specifically, the view that individual ‘minds’ represent knowledge of an objective world. On that view, knowledge production becomes a social process that depends on individuals and, above all, minds and/or brains. Influentially, Drucker (1969) related this to the economic concerns of a ‘knowledge economy’. The concept took on new functions which Gibbons et al.’s (1994) coinage of ‘Mode 2 knowledge’– ways of knowing that aspire to achieve social, political, or economic advantage. For Ziman (2000), post-academic science separates knowledge from ‘disciplines’ and, in their stead, seeks validation from industry, government, or society. In such a usage, the focus is, not the individual, but the process. Below, however, I follow neither Descartes nor Drucker. Rather, like Giere (2004), I treat human intelligence as necessary to making, revising and maintaining *all* scientific knowledge: science and peer-review are paradigms of cognitive activity. Human cognition is thus defined as that which enables flexible, adaptive behavior.

The paper leaves aside debate on the origins or nature of human intelligence. Rather, it regards organism–environment relations as the likely basis for all knowledge making. Broadly, this is embodied cognition (for an overview, see Shapiro, 2010); however, in asking how peer-review serves science, I stress the transformational role of cultural objects. The paradigm cases become, for example, how people, say, bring a ship into port (Hutchins, 1995) or design, build, operate, and construe output from the Hubble telescope (Giere, 2004). On this systemic perspective,^4^ reviewers drive the recursive activity of peer-review by scrutinizing a document’s images, texts and data (later, called ‘symbolizations’). Cognition is as social as it is individual: intelligent decision-making arises in the multi-scalar coupling of brain, body, and world. While much depends on platforms and computer hardware, scientific expertise serves in making and evaluating cultural products. The power of a publication lies in, not just materiality, but how people use inscriptions data and graphics to connect up experience, language, and culture. Language is thus traced to, not just brains, but how people coordinate action with both text and a history of making and hearing articulatory and gestural movements. Though based in bodily activity, writing-systems grant language a new historicity. As a result, language is, at once, embodied and amenable to description as verbal pattern (indeed, it is often confused with such patterns).^5^ The symbiotic nature of language activity ensures that, like navigating a ship or using the Hubble, peer-review is a distributed cognitive process. In the terms of Hollan et al. (2000), such processes are social, draw on artifacts and, above all, how cultural products can transform *later* behavior. Language – and document co-construction – links neurophysiological dynamics with judgements about the sense of perceived wordings. On this distributed perspective, far from being an inner system (or code), language is defined as *activity in which wordings play a part* (Cowley, 2011, 2014)^6^.

Even reading uses anticipatory saccading (see, Järvilehto et al., 2011): though neurophysiology is needed, historicity enables persons to link movements, interpersonal experience, and verbal pattern. While embodiment ensures the ontogenetic emergence of language, many practices are dominated by digitized patterns. In the practices of peer-review, inscriptions aid all parties in assembling descriptions of collecting, cleaning and processing data, reporting results, and discussing findings. They connect up graphics, inscriptions and electronic data such that, ideally, a reader could replicate what is described. Since persons make, construe and transform documents, peer-review is *more* than a normative institution. From this systemic perspective, peer-review is reframed as enabling and disabling knowledge making. Accordingly, it is hypothesized that:

- Multi-scalar recursive activity connects knowledge claims with what a community are likely to accept. Among other things, peer-review draws on:
  ◦ Using agonistic evaluation to create, constrain, and delimit how symbolizations are to be configured (and claims made).
  ◦ Recursive re-embodiment of symbolizations that reformat a document as (a) conforming to standard views of topics/debates; (b) narrowing and/or moving beyond a problem space; and (c) introducing contingencies that lead to unexpected changes in the final product.
  ◦ Prompting authorial change in, for example, (a) argument structure; (b) knowledge claims; (c) presentational style; and (d) the selection of wordings. Changes appear as symbolizations are replaced, revised, retained, and reconfigured.

Before pursuing the hypothesis, I offer an overview of the dominant approach while drawing heavily on Bornmann’s (2011) work. In so doing, I evaluate the ‘object’ of peer-review by linking his findings to a wide range of approaches that treat peer-review as a kind of ‘quality control’. Having sketched a consensus view of the state of the art, in section “Other Framings”, I turn to dissenting views and use these to place peer-review in a cognitive frame.

### Peer-review: The Semi-official History

**FIGURE 1:**
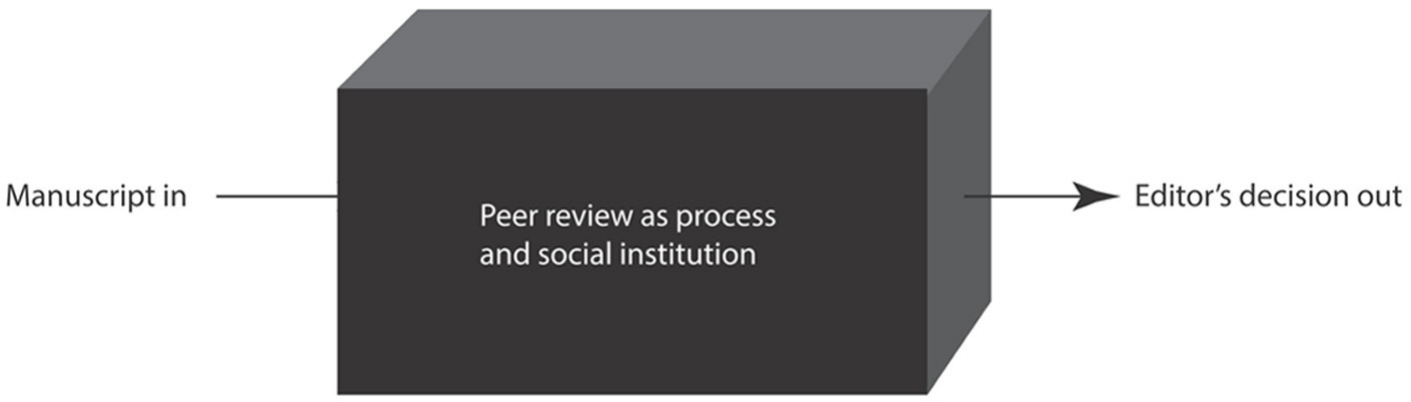
**An input–output model of peer review.** When authors are seen as offering ‘input’, peer-review becomes an institutional decision-making process that connects and editor, referees, and an electronic platform. The decision can be seen as ‘output’, and peer-review becomes a black-box.

Merton (1942) ensured that editorial peer-review came to be seen as a normative process. Not only does his view frame Bornmann’s (2011) approach but, among others, it grounds Ziman’s (2000) account of *science* and Huutoniemi’s (2015) recent encyclopedia entry. On this model, a manuscript is input that is evaluated to reach an output or a decision for publication or rejection (**Figure 1**). As a standard social practice, peer-review serves an institutional function in a ‘middle world’ (Merton, 1942). Science is idealized as accumulating potentially true and secure knowledge based on CUDOS values –Communalism, Universalism, Disinterestedness, and Organized Skepticism. Today, disinterestedness is underplayed and, as in Merton’s later work, some insert the criterion of ‘Originality’: like Huutoniemi (2015), many echo Popper’s critical scrutiny of ‘knowledge claims’ and treat organized skepticism as defining scientific practice.

Peer-review is often traced to the Enlightenment (e.g., Godlee, 2002; Ioannidis, 2005; Bornmann, 2011; Smith, 2011; Park et al., 2014).^7^ Wikipedia is not alone in, perhaps inaccurately (Ravaud, 2015), attributing the ‘first recorded’ pre-publication peer editorial to the Philosophical Transactions of the Royal Society.^8^ Assuming its enlightened nature, many stress that the ‘process’ was institutionalized after the Second World War. In that it reduces peer-review to changes in ‘form’, appeal to institutionalization is anything but trivial. There is, of course, dissent; for Gould (2013) peer-review arose from ‘censorship and inquisition’ (Gaudet, 2014) and, turning from a process view, while Hirschauer (2009) stresses reciprocal accountability, Gaudet (2014) underlines its bounding function. For Pontille and Torny (2015), as a technology, peer-review ties evaluation to aggregated epistemic judgements (validation). Before pursuing its role in knowledge-making, I present Bornmann’s (2011) view of how peer-review appears when pictured as a social process used in academic evaluation or quality control.

### Evaluation

By presenting peer-review as enlightened, its own ideals can be examined against normative categories. Bornmann (2011) asks if decisions are reliably obtained, free of bias and if the results have predictive validity. He echoes, for example, Reinhart’s (2010) contrast between ‘quality assurance’ and ‘self-regulation’. On the input–output view, process-variations (e.g., pre vs. post publication review) name independent variables that ground hypotheses and models. Investigations can thus build on constructs like efficiency (operationalized as time-taken to review) and effective selection (operationalized by measures of quality).

Since the 1980s, a consensus has arisen –as shared by, for example, Bedeian (2003), Hirschauer (2009), Bornmann (2011), and Gaudet (2014). Building on empirical extensions of Merton’s (1942) work, this is deeply influenced by biomedical concerns. In large part, this is due to the importance of sound evidence in health science and how, since 1989, the *Journal of the American Medical Association* (JAMA) has run conferences on the topic.

Where science is taken to advance in a linear way, progress is ascribed to consensus. In reviews of quality, one expects consistency; if reviewers were ‘fair’, outcomes would be psychologically *reliable*. However, this is not so: in a well-known example, Ernst et al. (1993) sent a paper submitted to a medical journal to 45 experts. When checked against chance, inter-rater agreement fell in the range of 0.2–0.4. Although, generally, there is more concord in recommendations for acceptance than rejections (c.f., Weller, 2002), reliability is poor across *all* such studies (Bornmann, 2011). Further, consensus is, at best, partial; few even ask about assumptions made, theories used, or methods pursued. Moreover, the ‘unreliability’ of peer-review applies to the natural sciences, the humanities and social sciences. For Marsh et al. (2008), lack of acceptable agreement among independent assessors is the major weakness of peer-review. Some are not surprised. Lack of consensus can be traced to diverse positions, backgrounds, criteria of judgment and, occasionally, dissensus is valued (Bailar, 1991). Finally, some think that a quest for reliability posits the wrong object of study (Gaudet, 2014) and/or a distorted view of science. In any case, ‘good’ reviews, whatever those may be, are highly unlikely to be reliable.

Peer-review features many kinds of bias. Indeed, Simon (1947, 1991) challenged Merton’s (1942) idealized view of reason by showing that individual rationality is bounded. Building on this, Kahneman and Tversky (1979) made bias central to the psychology of decision making. Heuristics can be shown to ground expertise both in the lab and elsewhere: while much is now known about biases, heuristics and natural decision-making (Kahneman, 2003; Klein, 2008; Thaler and Sunstein, 2008), peer-review ignores such work. Where bias is studied, this is usually in a loose or colloquial sense of the term: Bornmann (2011) counts 25 attested biases and reports no studies of how biases improve peer-review. He shows that work may be undervalued when, for example, reviewers draw on extraneous factors like perspective, provenance, gender, etc. A ‘halo effect’ appears when bad reasons (e.g., cronyism) lead to over-valuation. Building on Cole et al. (1981), most accept that, in reviewing large grants, a chance element occurs. Further, even bias is systematic: Bornmann (2011) shows how, at times, gender bias is marked and, at others, disappears. Further, once reduced to a variable, it is unclear if gender reflects lexico-grammatical markers (e.g., names and pronouns) and/or how women/men write manuscripts (and their parts).

Not only do reviewers fail to identify most inaccuracies but they rarely identify malpractice (e.g., Ioannidis, 2005; Smith, 2011). While changes in detection render it hard to quantify, as *Retraction Watch* shows, fraud is on the rise.^9^ Given the rewards, cases are frequent in the biomedical sciences where unethical conduct may be especially damaging. For Pontille and Torny (2013), malpractice includes selective and positive bias in reporting results, plagiarism (and self-plagiarism), guest and gift authorship and, indeed, data falsification. For example, in 2012 the Japanese Society of Anesthesiologists obliged Dr. Fuji of Toho University to retract 172 (sic) articles that used falsified data. Remarkably, Pontille and Torny (2013) find that Dr. Fuji’s work had been questioned as early as 2000 when a meta-analysis of 47 articles on anesthesia showed ‘remarkably identical frequencies of headaches as side effects’. Like conflicts of interest, fraud is largely invisible to peer-review. Further, in cases such as working in pharmaceuticals, academic research meshes with commercial interests (see, Mirowski, 2011). Among its many dangers are those of hiding data, focusing on positive results (e.g., Friedman and Richter, 2004) and, of course, allowing pharmaceutical interests to support journals as Elsevier did (Hansen, 2012; cited in Pontille and Torny, 2013). As ‘quality assurance’ peer-review is unfit for purpose.

In treating peer-review as normative, emphasis falls on an input–output process. Leaving knowledge aside, weight is placed on how Merton’s (1942) CUDOS values (or similar) are brought to bear on data and images and texts to give rise to a computer-like decision process (see **Figure 1**). While predictive validity may be a desideratum for grant applications, oddly, editorial peer-review is often seen the same way. For example, Bornmann and Daniel (2008) show that 95% of papers rejected by *Science* later appeared elsewhere. Leaving aside the journal’s status, they find no generally valid rejection criteria. Often, rejected papers are later accepted by journals of ‘higher’ standing (Weller, 2002). Others seek to validate peer-review with citation counts. While finding only five such studies, Bornmann (2011) reports a ‘rather high degree of predictive validity’ in using citation to assess editorial decisions. Yet, since these apply to a few Journals, the results conflate a paper’s quality with a journal’s perceived reputation. As Bornmann (2011) notes, high citation rates are likely to correlate with a Journal’s visibility. Further, questionable practices appear: in a study of journal ratings Pontille and Torny (2010) treat the method as a scientific apparatus that also serves as a political instrument. The field’s difficulties arise from the goal of defining criteria that grant ‘quality’ to –not a published paper –but a journal. In practice, appointed bodies use three sets of criteria: first, the journal’s procedures must meet international standards of, above all, peer-review. Yet, to establish Journal ratings intrinsic definitions (e.g., how journals are seen in a field) must, somehow, be squared with relational counterparts (e.g., an acceptance level below 25%). Yet, for knowledge what matters is, not the journal, but scientific content. And, by any epistemic criterion, few publications have *any* impact at all^10^.

### Other Framings

Not all treat peer-review as assuming a ‘linear understanding of progress’ (Ochsner and Hug, 2014). Indeed, Ziman (2000) sees unrealistic views of scientific method as part of the Legend and, rejecting such ideas, Bedeian (2003) turns to social constructivism. However, if science is heavily influenced by contingencies and thus non-linear, one can hardly be surprised that peer-review is unreliable, subject to bias and weak in predictive validity. How, could reviewers identify the future impact of the unexpected (especially where indicators are weak)? Much the same applies to Mode 2 knowledge and post-academic science: where focused on, say, social legitimacy and transdisciplinary concerns (e.g., Lee et al., 2013; Huutoniemi, 2015), one is bound to expect some reviewers to overlook what others see as pressing contextual and societal concerns. There are reasons not to address – not only how interactions shape reviewing – but also who counts as a ‘peer’ and how issues vary across occasions and genres of review.

Socially motivated criticism offers no alternative to the input–output or process model. The sharpest challenge is, perhaps, Pontille and Torny’s (2015) use of French pragmatic sociology. Rejecting rationalist models, they build on Latour (1987) to treat peer-review as a *technology* whose practices/artifacts co-evolve in the social world. Rejecting mid-twentieth century ideals of science, they present two ‘tests’ or modes of performance-control. For Pontille and Torny (2015), peer-review uses practices or assessment procedures (evaluation tests) that are necessarily in tension with criteria pertaining to the quality of text, data and images (validation tests)^11^.

### The Academic Dissenter

In treating peer-review as a normative process that is amenable to quantitative investigation, experts concur with their negative verdicts. Peer-review is unreliable, biased and lacks predictive validity. Yet, the ‘process’ not only takes considerable labor, but it also shapes the goals, practices and beliefs of aspiring academics. So how do professionals experience peer-review? The question is rarely asked and, suprisingly perhaps, surveys tend to be positive; in Bornmann’s (2011) view, good stories outnumber the bad. Authors learn from reports –however, unreliable and biased they are. Indeed, process models may contradict common academic experience because they fail to identify the *de facto* functions of reviewing. Below, I rethink review reports as cognitive resources: they set enabling/disabling conditions whose tone and content influence the author’s response. Though normative, like all social practices, the structural and interpersonal dimension of peer-review unite convictions, expert (and other) biases and social change. As human activity, peer-review varies dramatically between fields. Further, just as technology changes, so may peer-review; habits matter in science too. Scientists develop new ways of working, new thinking and new products –they develop new kinds of community.

## Seeking A Larger Frame

Approaching peer-review as quality control is wanting. Much can therefore be gained from reframing the normative social process. In part, this is because post-academic science builds on values that contrast with Merton’s (1942). To twenty-first century eyes, it is striking that scientists were once seen as disinterested observers who were rewarded for selfless acts. As respected people, they dutifully sought to replace war-time horrors with a new society. Scientists were well-paid, securely employed and had academic freedom: they were aided by secretaries, undertook laboratory tasks, read, and discussed ideas with students. No-one pushed them to publish, attend conferences or scramble after grants. Later, “[T]he image of an upright reviewer gave way to one of a colleague steeped in self-interest, prejudice and beliefs which formed an integral part of his/her opinion” (Pontille and Torny, 2015, p. 65). Given social change, in discussing CUDOS values, disinterestedness is often overlooked. What of the other values? Is organized skepticism challenging? Is it merely checking for conformity? This matters if, as for Huutoniemi (2015), its focus is on ‘knowledge claims’. For, while ‘quality’ and ‘originality’ are often invoked, there is little evidence that these can be identified. How many highly cited papers are worth reading? Are they correlated with prestige (and high impact) journals? As the case of peer-review shows, science can produce correlations, opinions and valid and yet, unclear, results^12^.

Mats Alvesson brings just this logic to his field of organizational science:

“This article aims to place the question ‘do we have anything meaningful to say?’, more strongly on the agenda when carrying out and assessing research. I start by pointing out that we, as a community, often have little to say to anyone outside a small group of like-minded academics. … Much of what I am saying here may be less relevant for those struggling to get their bread and butter, and whose overriding concern may be about their CV and publication record in order to secure a position—and identity and status as someone who is a ‘real’ academic. But … most people should be concerned with more than just landing and keeping a job or experiencing acceptance and membership in academia; and thus having something to say should be vital” (Alvesson, 2013, p. 79).

Science changes as much as scientists –in my view, readers ought to be shocked that leading voices proclaim that many have nothing to say (and that influential Journals regard the work as worthy of publication). However, in turning from the secrets of art and nature, this is perhaps less surprising. After all, science increasingly focuses on social practices such as peer-review. In offering academic status to proponents, such a topic can also be seen as scientific mission creep –as a way of leaving aside foundational issues in the name of social and contextual results. While opinions and correlations show, repeatedly, that peer-review fails quality control, the normative framing has not been scrutinized. This is a scandal because, as every social scientist knows, *all* social practices have this status. It is trivial to call peer-review normative – the same applies to shopping, electioneering, bathing and mourning. In spite of lip service to organized skepticism, few have considered the assumptions, set up debate or sought to challenge input–output logic.

### Contexts of the Non-crisis

Peer-review fails its own *desiderata*. If it does not function as quality-control, is it a mode of censorship? Is posing such a question a sign of crisis? While not offering answers, some insight is gained by asking why the field became self-preoccupied.

With globalization, universities in the US, UK, Germany, and other Western powers ceased to control knowledge production. New views of knowledge led to the politicization of academia as business interests and national governments sought to defend ‘their’ knowledge makers. Money went into rankings purporting to measure *excellence*. By treating peer-review as quality control, bureaucrats offered ‘independent’ justifications for the uneven resourcing of institutions (e.g., in the UK), claiming the reality of knowledge transfer (e.g., in the European Union) and the rise of global excellence (as in China). Especially in the US, the UK and Australia, political change transformed universities into a service sector financed by student customers. In *Science Mart*, Mirowski (2011) paints a shocking view of how research is outsourced, separated from teaching that is, increasingly, performed by low paid, junior staff. In the UK, the Blair government linked public policy with research practice. Using a 1999 White paper, *Modernizing Government*, research was funded to generate evidence supportive of government goals and policy. Further, a series of Research Assessment Exercises served to “concentrate funding on a small number of research organizations judged to be successful on… [its] internal criteria, causing the weaker ones to lose their better leaders and staff and undermining their access to the human and financial resources that will enable them to improve their performance” (Boaz et al., 2008, p. 246). Under such pressures, it is to be expected that “some domains of science tend toward collaboration with power elites” (Huutoniemi, 2015). Indeed, I left the UK partly as a result of pressures created by the Research Assessment Exercise (RAE) and partly because I choose to work with students as, not customers, but co-constructors of understanding. As peer culture disappears (Reinhart, 2010), gentlemanly self-regulation is often replaced by performance driven roles. Indeed, the globalization of knowledge is itself enough to ensure that science is no longer dominated by men of a certain class who meet in the smoking rooms of London clubs –or their 20th century counterparts (see, Snow, 2012). Changes in society, science and scientists and an innumerable array of related practices ensure that peer-review has accrued new functions. To cite just one set of examples, not only does ‘impact’ influence careers but many choose to focus on, not a discipline, but television appearances, consultancy positions, or coining buzz-terms (e.g., Mode 2 knowledge). In social psychology, there may be a tendency to seek out the ‘counter intuitive’ (Shea, 2011). Thus, Diedrik Stapel’s papers suggested that Alpha females tend to philander and that having a messy desk can indicate racism. In these cases, however, scandal broke; an academic had used invented data! Not only does fraud elude peer-review but, for Shea (2011), ‘addiction to surprising findings’ characterizes high impact journals; he cites, among others, a paper in *Psychological Science* that ascribes better decision-making to people with full bladders (Tuk et al., 2011). While not challenging the work, Shea (2011) finds the claim overstated.^13^ There is a paradox: although peer culture is disappearing, even re-engineered institutions rely on peer-review and publishing.

Technological change shapes new practices. Just as the 1960s invention of Xerox transformed peer-review (once one could copy manuscripts, they could readily be posted to reviewers), the internet has driven change (e.g., reviews can be managed electronically; measures can be made of impact). Given such results, many universities seek to measure academic ‘success’ by citation indices. Where counts are purely quantitative, reputation ceases to be linked to prestige journals and, for this reason, publishers feel threatened. Following their use in Australia in the 1980s, vested interests shifted the focus to Journal ranking (for an account, see Pontille and Torny, 2010). A similar compromise between political economic players shaped the UK’s Research Excellence Framework (REF). Although all submissions presented papers for peer-review, this was a token move: universities admitted or excluded researchers on the basis of journal citation indices. Bizarrely, journal ‘quality’ became a proxy for scholarly ‘quality’. Not only did REF privilege some institutions but its indices were biased to established views. In such a system, Mertonian ideals serve powerful elites who gain from maintaining the myth that peer-review is to be seen as disinterested, reliable, unbiased, and valid.

Vested interests need to invoke quality control. However, new kinds of peer-review and academic products are also challenging the establishment. Increasingly, reviewers are paid (see, Pontille and Torny, 2015) and some have experimented with open peer-review, post-publication review, and crowd sourcing. Many editors challenge orthodoxies. Various academic practices have transformed or abandoned. While the biggest changes may lie in library use and reading, others are easier to document. First, given demand for citations, publishing is much faster. Second, journals have on-line portals, offer pre-publication services and provide databases that prompt access to publishers’ other journals. Third, authors are rarely offered hard copies of publications and, in many cases, editorial decisions are made with reference to markets (For example, when a new manager was appointed, Elsevier encouraged (or pushed) 3 of it 4 Linguistics editors to retire). Fourth, open access has become a scientific and political issue that has led to the proliferation of new journals. Though so-called predatory journals attest to how many papers lack epistemic value,^14^ many new journals make striking contributions. For example, since its launch in 2006, PloS One has become prominent. Instead of calling for comments on manuscript scope and innovation, it publishes on the basis of technical review. As an ‘evolving platform’ PloS One stresses, not claims of originality, but thorough science. As a result, the platform publishes more articles than anyone else (31,500 in 2013) and, using social networks, reaches non-academic circles. Thus, while a normative social process, peer-review is *also* (part of) a socially distributed cognitive technology.

### A Clear Picture

Recognition that peer-review does not function as effective quality control is inseparable from a social, academic and technological context. Yet, power elites rely on the Mertonian image. Not only does it legitimize policy moves but as Fitzpatrick (2011) notes, peer-review serves ‘institutional warranting’. However, that is only a part of the story. While evidence is weak, many (or most) academics value peer-review. Far from being because it is a normative process, this is likely to bear on their experience of its results. Thus, for Squazzoni et al. (2013), it is crucial to ‘knowledge generation’ and, for Huutoniemi (2015), it can be ‘assumed to guarantee good science’. Peer-review may be a cornerstone of knowledge production.

While peer-review may grant scientific status to documents that are flawed or, indeed, merely review opinion and compile correlations, it serves many scientific, social, and political interests (see, Readings, 1996; Mirowski, 2011). It may seem, therefore, that peer-review is safe; reformers can polish its image with expressions of concern and promises of change. Nonetheless, peer-review performs poorly as quality control; nor does reform address any dissonance between experience and expert views. I now argue that, once one abandons a 1940s ideal of science, peer-review ceases to reduce to a social institution that depends on input–output. Instead, I use systemic cognition to reframe peer-review as using a social technology whereby all parties collaborate to manipulate texts, data and images or what will be termed ‘symbolizations’. Given the power of the latter, I claim, peer-review has an important role in *making* scientific knowledge.

## Systemic Cognition: The Basis Of Science

**FIGURE 2:**
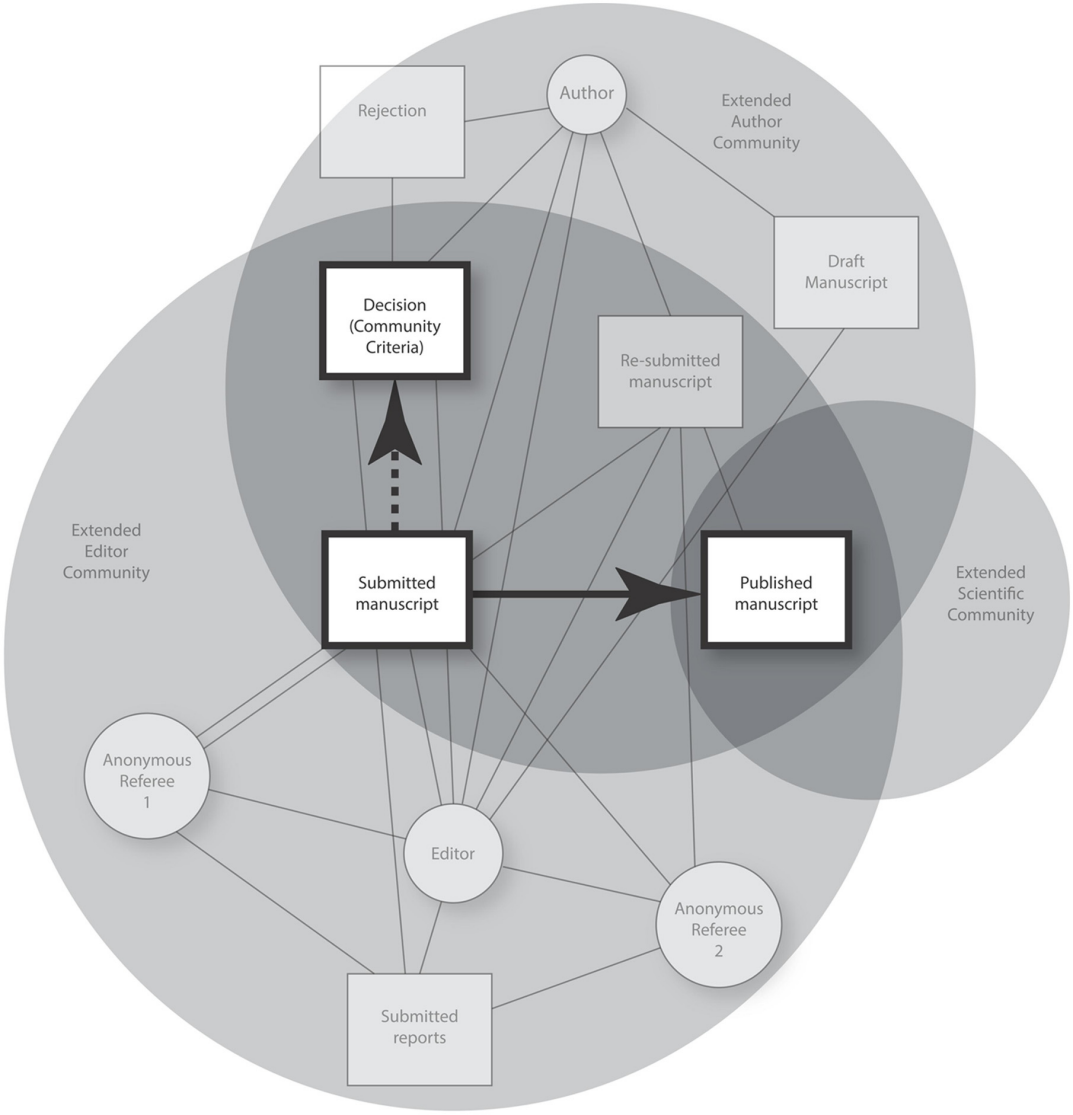
**A systemic model of peer-review.** The model places author, editor, and referees in overlapping networks and structures. The author’s recursive re-embodiment of a manuscript connects up thinking, less formal communication, and its more formal counterparts. Where seen in input–output terms, the focus falls on how a manuscript is evaluated (see, large stippled arrow). As systemic cognition, by contrast, peer-review constrains the activities connecting the submission of a ‘successful’ draft to its eventual academic publication (see, unbroken black arrow).

By definition cognition enables flexible, adaptive behavior. It enables people to undertake complex projects by using institutions to engage with each other and kinds of equipment. On a systemic view, the sociology of science connects with distributed cognition (see, Magnus and McClamrock, 2015). There is, as Robert Giere (2010) emphasizes, a parallel between science and agriculture: above all, in both kinds of practice, a multi-party, multi-agent system generates output. However, whereas farmers produce potatoes, wheat or coca leaves, scientists read, undertake experiments and aggregate data, graphics and inscriptions (‘symbolizations’). Those concerned strive to improve outcomes in ways that, in agriculture, as in science, draw on social conditions and the use of cultural products. Individual activity occurs within domains such as farms and universities or what Hutchins (2014) calls ‘cognitive ecosystems’. On this view, one can look beyond the normative by allowing peer-review to include Gaudet’s (2014) ‘structural relations’ and Pontille and Torny’s (2015) ‘technology’. Using these models, **Figure 2**, presents the two contrasting framings of peer-review. The black arrows show both process (input–output) and systemic views of its function –placing them against a background of scientific networks.

Publication renders text, data, figures, and other features of a document available to knowledge makers in a scientific field. Importantly, this can influence both the reputations of those concerned and later readings of the published copy. The figure thus presents extended communities of authors, editors, and science in general. The arrows represent idealizations: as a social process, peer-review functions to map a submitted manuscript onto a decision (framed by community criteria). By contrast, as systemic cognition, peer-review leads to, not just a reviewer’s decision, but (in some cases) rewriting and a published product. By analogy with agriculture, just as a farmer grows crops, if of epistemic value, this can nurture a discipline or field. Publication is a cognitive *event* that, for a field, marks a ‘change in the layout of affordances’ (Chemero, 2000): it brings forth new knowledge and opportunities for knowledge-making. Ideally, one would examine how author, editor, and reviewers collaborate to shape the manuscript. For current purposes, however, I focus on method, the role of data, inscriptions, graphics, etc., and, in illustration, illustrate how one can address three hypotheses.

### Preliminary Remarks on Method

As applied to peer-review, the functional approach of cognitive science can pursue: (1) What is peer-review for? (2) How does peer-review work? Within a systemic frame, it is seen as allowing people to collaborate in reformatting a manuscript that can influence knowledge making. While quality control remains a desideratum, reviewers are *also* concerned with disciplinary (or transdisciplinary) knowledge. Of course, the process depends on values that reflect, for example, whether parties aim at reaching academics and/or non-academic stakeholders. For now, however, I leave aside issues about science in society to focus on basic implications of the model

(1) Authors, editors, reviewers and the wide public engage with publications (and pre-publications) as members of partly overlapping, partly competing communities.
(2) Even if a pre-publication is submitted electronically, the editor selects referees, mediates between parties (including ones not shown in the figure) and makes/communices decisions–as well as formulating views and accompanying advice. Peer-review is dominated by interpersonal contacts.
(3) Referees link the material to what expertise they have and their grasp of a journal’s aspirations. They then submit reports (perhaps following guidelines).
(4) The editor evaluates the reports in making a decision/recommendation. Normally, the editor sends both reports and accompanying advice (e.g., what to prioritize) to the authors.
(5) Where a manuscript is not rejected, an author will evaluate reports and advice and, on this basis, decide how to proceed (and whether to resubmit).
(6) Rewriting will draw on discussion with co-authors and others in his or her community as well as published sources (or doing experiments) suggested by referees.
(7) Once resubmitted, a manuscript sets off a cascade of events. (While the diagram shows an accepted resubmission, a document may be sent for further review by the same or different referees). There may be several rounds of resumbission and re-evaluation.
(8) Once published, a manuscript is amenable for dissemination. Alongside informal appraisal, this will affect the reputation of the Journal, the editor, and author (where referees are public, they too will be affected). In slow scales, this will alter future reviewing (and, especially, the selection of reviewers).

Far from being sequential, peer-review is multi-scalar cognitive process. Its recursive evaluations link individual knowledge – and literacies– with cultural products that realize (or fail to realize) a community’s values, knowledge, beliefs, assumptions and standards. Indeed, ‘skepticism’ applies to more than knowledge claims. On a systemic view, a research program would link such matters with both the anticipatory nature of cognition and how, over time, knowledge claims are interpreted. For now, however, I assume that that a manuscript has already been submitted.

Since peer-review is multi-scalar, change arises between manuscript submission and, when accepted, final publication. Pre-publication events thus play out recursively around the solid black arrow in **Figure 2**. Later, I discuss three sub-hypotheses: (1) reviewers use, not organized skepticism, but agonistic evaluation; (2) recursive re-embodiment enables referees/authors to negotiate or demand conformity to standard topics/debates, presentations of a problem space, and, at times, allows contingencies to transform the product; and (3) rewriting can alter, among other things, argument structure, knowledge claims, presentational style, and choices of wordings. However, before addressing such questions, one faces a challenge. The many activities that contribute to peer-review have to be matched to a published outcome. It is necessary to bring the linguistic and non-linguistic activity of authors, editors, and reviewers together. Plainly, this is irreducible to the construal of data, images, and propositions into which a text can be analyzed: just as skilled farmers produce crops, the expertise of literate scientists shapes the published product.

### Condensing Sense-making

On a systemic view, cognition centers on a project (e.g., how a ship is navigated or how science uses the Hubble). In focusing on such achievements, what happens does not reduce to individual knowledge, skills and beliefs. In peer-review, therefore, one must consider what happens to the data, inscriptions and graphics (symbolizations) that appear as a document. While a computer metaphor attributes processing to mental states that co-vary with physical structures, this philosophical view offers little to the study of distributed cognitive systems. In spite of a conservative view of language (see, Steffensen, 2009), Clark (2008) allows thinking and gesture to involve objects that contribute to believing, doing and perceiving. Even Aizawa (2015) allows that bodies contribute to cognition in ‘surprising’ ways. While the nature of embodiment is a current topic of debate (see, Shapiro, 2010), its role in human intelligence is beyond dispute. Indeed, on a radical view, agent-environment interactions shape all cognitive events (e.g., Chemero, 2011). For current purposes, however, it is enough, first, that neither minds nor brains depend entirely on inner stores of data; second, that language is irreducible to a code (e.g., Love, 2004; Cowley, 2011). In today’s terms, while embrained, human thinking is also embodied, embedded, enacted, and extended: bodies rely on attuning to each other and the world. Language too arises as people embody their dealings with each other, objects, and social practices. Over time, as speaking/hearing beings, agency develops as they become persons who orient to beliefs, doubts and knowledge. Thus, while individuals rely on certainties, *facts* are crucial to human form of life.

In science, knowledge is both social and inseparable from practices and beliefs. Thus, in laboratories and libraries, cultural ecologies (Hutchins, 2014) have a powerful influence on how people feel, think and act. Perceiving an utterance-act draws on – not just facts – but how physics shape its likely role in coordinated action (Cowley, 2014). Thus, for example, the physics of ‘Boo!’ or ‘That can’t be true’ affect how such an utterance act is evaluated. More strikingly, perhaps, reading is anticipatory as attested by measures of fixation-speech intervals (Järvilehto et al., 2011) While the embodied nature of languaging –and language –is increasingly studied (e.g., Linell, 2009; Thibault, 2011; Steffensen, 2013) less attention has hitherto been given to how linguistic practices can *sustain* knowledge, attitudes and beliefs. In treating peer-review as just such a practice, I build on Kravchenko’s (2006, 2007, 2009) bio-cognitive approach. Like Linell (2004), he contrasts evanescent speech with the perduring nature of written marks. However, turning from the distributed and the dialogical, Kravchenko (2006, 2007, 2009) focuses on the ‘symbolizations’ that come to constitute new kinds of understanding. As further explained below, this is because, unlike acts of speech, visible patterns can be preserved in manuscripts or, indeed, transmitted in stable and compressed form (as digitized code). Accordingly, the ‘same’ patterns can appear under many perspectives and be evaluated in many contexts. In pursuing this view, first, he contrasts talk and literacies (e.g., sending text messages or reviewing manuscripts) and, then, he stresses that human Language (with a capital ‘l’) glues together social practice.

Kravchenko (2007, pp. 662–663) uses Russian tradition: “language is an activity that involves all the functions which make humans human. And language is an activity that generates the means for its realization in concord with the diverse functions possessed by language” (Zvegintsev, 1996, p. 50 Kravchenko, 2007). By extending the ecology, Language connects speech, attention and gesture with solo and collective modes of action. As peer-review uses literacies (and other capacities), the practice makes extensive use of inscriptions. People construe manuscripts against individual experience by drawing on Language to evaluate data, graphics and inscriptions. In peer-review, application of *symbolizations* can thus be extended to digits, quantitative data and pixelated images. While unlike inscriptions in that, for example, images cannot be ‘read aloud’, these all serve as replicable constraints (Raczaszek-Leonardi, 2012); their perduring nature transforms collective memory (Donald, 1991). Symbolizations evoke Language that, like a living system (Kravchenko, 2007), enables persons to fine tune actions, perceptions, thoughts, and beliefs. Humans act and think partly under collective control (see, Raczaszek-Leonardi and Cowley, 2012), or, for Kravchenko (2007), “Once we view society as a unity of interactions, we see the crucial sustaining role of a linguistic ecology.” In science, symbolizations define frameworks, assumptions, procedures and ways of measuring; as they perdure, take on what Craik (1943) terms *objective validity* (e.g., *E* = *mc*^2^). However, while Craik (1943) appeals to the brain, the bio-cognitive alternative emphasizes a history of recursive coordination that, in Maturana’s (1978) and Maturana and Varela (1980) terms, allows people to develop consensual domains and social communities. Over a history of co-ordinations, people come to *observe* what is said and done: observations of actions (e.g., measures) enable scientists to evaluate knowledge claims. In peer-review recursive activities connect expert experience, symbolizations, and observations. As with cities, laws and local customs, symbolizations link normative practices with a kaleidoscope of senses. While bounded in rationality, people draw on laws, architectures and verbal patterns. Like institutions, these appear non-temporal (a person revisits an arrangement of letters, a legal judgment or a dwelling) as people glue together domains of human life. In *theoretical culture* (Donald, 1991), symbolizations dominate modes of action as diverse as, say, legal, religious and scientific practice.

While some call symbolizations ‘external representations’^15^ Kravchenko (2009) argues that, even if they are stable, symbolizations evoke connotations. They index, not just denotata, but also *individual* experience. In illustration, he turns to the etymology of συμβoAAAoν, symbolization, an inscription that alludes to how ‘marks’ are ‘thrown together’. In a world of Language, the use of writing-systems can aggregate cultural products. Far from relying on coding (or invariant propositional knowledge), individuals link symbolizations with both usage and their own experience (e.g., of data analysis). Although symbolizations (and images) are empty, they perdure as anchors of experience (that varies across communities). In time, symbolizations are conventionalized, regularized, and contextualized to sustain social practices; further, they can be organized around complex historical practices and, as a result, granted a very precise sense.

In science, symbolizations enable the ascent of Mount Improbable^16^ or, in short, enable much knowledge-making. In simple cases, this is because procedures ground complex knowing. In algebra, for example, *x* + 2 = 7 is a symbolization of “*x* + 2 = 7”. In bio-cognitive terms, its sense depends on how an observer orients to Language. For a mathematician, it can be seen as a denoter that calls forth a rule of inverses: this ‘explains’ how a numerically literate person can see that it entails a *denotatum* symbolized by “the number 5”. Crucially, one can follow the procedure *without* ‘knowing’ the description. The denotation is equivalent to acting in accordance with a rule (seeing that *x* = 5). To come to understand why *x* is 5 is a considerable feat. However, knowledge making also draws on what Maturana calls connotations (see, Kravchenko, 2007). This can be illustrated in relation to uses of the equation, *E* = *mc*^2^. In physics, this identifies a mass-energy relation. Roughly, a denoter identifies a universal proportionality factor whereby equivalent amounts of energy and mass are equal to the speed of light squared. Yet, in a television studio, say, *E* = *mc*^2^ can be evocative: it may loosely suggest ‘intelligence’ or science. Indeed, as exemplified on the previous page, the symbolization can serve to evoke Craik’s concept of ‘objective validity’. Symbolizations condense connotations while connecting with a community’s denotata. This is possible because: (1) they draw on experience; (2) they perdure; (3) they allow many kinds of agreement consensus; and (4) in time, they generate conventions, arguments, procedures, and expectations. As a result, symbolizations warrant inferences and modes of coordination that reach far beyond ‘interpretation’. Finally, while Kravchenko (2007) limits use of ‘symbolization’ to ideographic and alphabetic systems, I extend the usage to digital and graphic ways of presenting data. Given perduration, symbolizations become replicable constraints that detach interpretations from a material substrate. Their non-local values contribute to writing, mathematics, music, maps and so on. Symbolizations like “*E* = *mc*^2^” allow diachronic functions and community-based knowledge. At any time, a symbolization can bring forth individual senses and/or hint at/specify *denotata*.

Since symbolizations are so much more than symbols (which, by definition, lack connotations), I now turn to how they contribute to the practices of peer-review and, thus, knowledge making.

### The Power of Symbolizations

Having presented symbolizations as replicable constraints that use writing-systems or algorithms to digitize data and graphics, I return to the systemic perspective on peer-review. In a cognitive frame, its multi-scalar activities are seen as allowing the recursive reformatting of aggregates of symbolizations. Accordingly, I turn to how a submission becomes a publication and, in section “A First Sketch of How Peer-review Constrains Cognition”, revisit the claims mentioned (viz., agonistic evaluation, recursive re-embodiment, and authors aggregate perduring symbolizations). Once seen as an epistemic process, peer-review is traced, in the first place, to how experts link their reading of a submission to a scientific community’s procedures and ways of using Language. As in censorship, peer reviewers fix what is *not* to be written (or published). Second, referees do not need much ‘understanding’ of the argument. Indeed, given perduration, symbolizations are tools or, in Maturanian terms, contribute to recursive activity in a consensual domain whose span reaches into the referee’s community. Where people orient consistently to values, they stabilize, facts, content and bundles of assumptions, metaphors and values. Much can be gained by aligning practices with symbolizations and, by so doing, binding normative activity, social technology and ways of legitimizing outcomes. This is possible, unlike speech, symbolizations draw on historicity; given relative invariance, they act as non-local resources that enhance induction, deduction and abduction (and thus grant ‘collective memory’). While such powers are embodied –and inseparable from affect –symbolizations connect up webs of knowledge. An aggregate of symbolizations thus evokes many ‘readings’ and modes of development. Given a history of recursive activity, an author can hone symbolizations by using referee reports to bring new criteria to bear on writing: a document’s content can be revised, reinterpreted and redefined. Peer-review thus serves both authors and wider communities. Documents shape structural relations between participants and, by using symbolizations, unexpected consequences are common.

Symbolizations have the power to change future knowledge claims. This is because, while frames become established, they are always contested. Symbolizations link impersonal expertise with individual experience: they can be read as conferring/denying status to theories, institutions and even persons. As Ziman (2000) stresses, what counts as scientific *knowledge* varies across disciplinary (and interdisciplinary) fields. If, in macro-physics, symbolizations hone law-based predictions, in other fields their uses are less readily defined. At times, indeed, dissensus may be more important than consensus. Indeed, this is precisely how I view the mainstream ‘object’ of peer-review research. While grounded 20 years of intensive research that treat it as a normative process*, it has become clear that peer-review is neither reliable, impartial, or the basis for predictive validity*. The italic symbolizations present hard won collective knowledge and, as such compress correlations and opinions that have been developed right across the field (e.g., Bedeian, 2003; Bornmann, 2011; Gaudet, 2014). They fix content or what, for peer-review ‘experts’, currently counts as *knowledge*: as such, even without so called ‘hard’ evidence, symbolizations delimit a problem space and, thus, define a likely empirical search space. By turning to the voice of academic dissenters, I propose a reframing of the study of peer-review that opens up epistemic questions. My suggestion relaxes these constraints by linking peer-review to the making of scientific knowledge.

### A First Sketch of How Peer-review Constrains Cognition

Since peer-review transforms documents, referees strive to influence how symbolizations are aggregated. As recursive activity, peer-review shapes what becomes collective memory. In framing this as systemic cognition, the method is illustrated by pilot work. Next, I describe review reports of a draft of this paper against three exemplary hypotheses:

(1) Agonistic evaluation is crucial to peer-review and the framing of knowledge claims
(2) Recursive re-embodiment enables referees to seek (or demand) conformity to standard views of topics/debates, familiar presentation of a problem space, and, at times, to set off contingencies that change the final product.
(3) Rewriting can lead to, for example, relatively fixed (a) argument structure; (b) knowledge claims; (c) presentational style; and (d) choices of wordings

As the author, I was struck by the reviews for two main reasons. First, in spite of my (unchanged) title, neither referee grasped the paper’s hypothesis –that peer-review constrains cognition.^17^ Second, neither commented on what I intended to be the paper’s main contributions. They left aside my reading of Bornmann (2011) and, generally, the literature on peer-review as well as the surprising claim that academic publications are, not texts (or sets of propositions), but aggregates of symbolizations. Accordingly, I focused these issues while responding to what I deem to be agonistic comments. In that these do not address knowledge claims, they do not represent organized skepticism: rather, both referees stress my failure to establish the paper’s main hypothesis. Their comments include:

Referee 1: “I am a bit troubled by the structure of your paper … ” I really had problems whether your paper was focused on issues of peer-review, or … “coordination between peer-review and cognition” … “the last paragraph is not clear” … “Provide more empirical support for this …”

Referee 2: “the writing is unnecessarily sloppy” … “I find it difficult to summarize the author’s own view” … “the author could be said to replace one strawman by another” … “there is a distinct and almost systematic lack of clarity in some places that could be considered a “fundamental flaw” …. This includes many “rhetorical remarks that are made in passing and that are not backed up by argument or reference.” … “too many, in my opinion, instances of vagueness” … “statements that are ‘hard to swallow’ …” I actually have no idea what is meant “cognition echoes Descartes’s dualist legacy and, today, the computational theory of mind” … [such] issues “are not as clear cut as they appear in this manuscript” … X is “another example of a statement that might be well received in talk in a pub” … “who is shocked by this?”^18^

They contest neither my reading of the literature on peer-review nor the reframing of what happens as recursive activity that leads to the re-aggregation of symbolizations. While not the place to document my response in detail, the comments evoke a negative affective valence. They prompted me to make many revisions. Specifically, in proposing changes in the tone or status of various passages, the reports set off a reiterated recursive process (the symbolizations of the referee reports focus my attention on aggregates of symbolizations that, when reread, induced me to reformat the aggregates of symbolizations in order to achieve less negative effects). This recursive re-embodiment forced re-engagement with my expertise –and my grasp of my aims –as I re-embody the writing. Though the results are amenable to propositional analysis as sentences and text, these derive from an affectively driven statement of expertise that re-embodies, and extends, my understanding. Of course, the referees do not ignore ‘content’; in this case, however, their concern was less with what I was proposing than an attempt to place it in relation to standard views of topics/debates and the current problem space of cognitive science. Strikingly (for the author) neither referee gave any weight to my claim that cognition is best defined in terms of the conditions that enable (and disable) flexible, adaptive behavior. Instead, I read:

Referee 1: Weight should be given to systemic cognition and ‘conceiving the action within and between systems’ as well as ‘offering description of the systemic process’ and ‘exploring the cited literature further’ as well as giving ‘empirical support to the types of analysis performed, context and limits of the evaluation and giving ‘detailed presentation of input–output models’.

Referee 2: Asks if it is important to establish consensus (saying that s/he may not be on the same page about this) … wants clarity about Descartes and the computational theory of mind … asks if it is true that computational models of ‘mind’ have been abandoned and points out that, in the extended mind theory, computation is still central.

In addressing such points, my resubmission neither reduces cognition to interactions within and between systems (rather, it focuses on what enables behavior) nor turns to the theory of extended mind. (No-one traces peer-review to a neural, or ‘mental’, domain). Rather, I chose to stress that symbolizations exploit, on the one hand, Language and, on the other, individual embodiment.^19^ Further, referee 1 says that s/he “expected a new formalization of the relationships between peer-review and cognition.” While at odds with defining cognition in terms of conditions that enable/disable the aggregating of symbolizations, this advice drove the paper’s most important changes. I explicitly contrasted the ‘normative’ input–output model of peer-review (**Figure 1**) with one presenting it as giving rise to an outcome based in recursive re-embodiment (**Figure 2**). Further, since neither referee commented on symbolizations I stressed that, unlike texts or discourse, symbolizations have a constitutive role in the making of scientific knowledge. While amenable to analysis as propositions, symbolizations evoke both Language and also individual experience. As part of normative social process (i.e., social behavior), they bind together interpersonal meanings, affect and impersonal norms. Given their relative invariance, they connect affect, orientations to the world, values and expectations. Like Ziman (2000), I explicitly ascribe scientific knowledge to groups; cultural ecosystems link statements and procedures with *objective validity* to many bundles of beliefs, values, rituals and modes of proceeding. Scientific knowledge is – necessarily –distributed: it cannot be ‘in’ symbolizations (or propositions) but arises as persons link replicable constraints to procedurally based experience. Seen as a cognitive ecosystem, peer-review is like using the Hubble telescope or acting to bring a ship into port: an author cooperates with others while reconfiguring aggregates of symbolizations. For example, changes affect: (a) argument structure; (b) knowledge claims; (c) presentational style; and (d) choices of wordings.

The resubmission has a more explicit argument structure (and is about 4000 words longer): the main structural changes are as follows:

(1) I drew a distinction between regarding peer-review as a set of practices that constrain cognition (by enabling flexible adaptive behavior) and hypotheses that depend on a bio-cognitive view of symbolizations (in the section, Cognition and Knowledge);
(2) I added new sections to present how a systemic view can be applied to peer-review and what this implies for method (in the section, Systemic Cognition: The Basis of Science and Preliminary Remarks on Method).
(3) I substantially revised the current section by adding a pilot study –showing that peer-review is cognitive as well as normative (in the section, A First Sketch of How Peer-review Constrains Cognition).

Ironically, while referee 2 claimed to be unable to formulate my view, I fully endorse his/her summary of the paper. I offer neither an argument nor a personal view of peer-review: in the referee’s terms, I see the literature as like meeting a series of ‘straw men’. Further, while peer-review is wanting as quality control, the referee correctly observes that I view peer-review as ‘knowledge generation’ and likely to ‘guarantee good science’. Like Ziman (2000), moreover, I regard good science as a real good (not a straw man) and, by way of emphasis, have extended my use of this perspective on *real science*. While the state of the art fails to establish the importance of peer-review, I have attributed this to its normative assumptions. It is likely, I argue, that science also enacts recursive social process. Indeed, authors link a changing draft with both their experience of Language and the referees’ reports: putting these together, they re-aggregate symbolizations. Further, given that I regard real science as important, I have *not* toned down my style. Far from seeing it as another straw man, I have added evidence and defended my claims.^20^ I endorse Ziman’s (2000) view that ‘value free’ science is a myth: values permeate both real science and its post-academic successor (perhaps, these are granted too prominent a role in the latter). Finally, regards choices of wordings, I focus on two issues: first, I claim (and repeat) that *cognition* can be defined around the enabling/disabling conditions of flexible adaptive behavior. Accordingly, peer-review is as social as it is individual –flexibility is due to more than neural or (‘mental’) agility. Second, I have increased the theoretical weight given to the concept of *symbolization*. Without perduring replicable constraints that mesh individual experience with impersonal patterns and shared procedures, I suggest that there could be no scientific knowledge.

Precisely because it results in aggregates of symbolizations, science is increasingly dominated by technologies. It is non-trivial that increasing use is made of internet platforms, rules and transdisciplinary encounters between editors, authors, and reviewers. Indeed, this clarifies why a person does not merely ‘write/review’ a paper or ‘perform/check’ experimental and statistical procedures. As Pontille and Torny (2015) stress, reviewers play the role of disclosing how they perceive content (e.g., the process model of peer-review), develop perspectives, offer evidence and get the author to engage with other views. Ultimately, social technology aids in shaping a body of coherent work –an aggregate of symbolizations that spreads across a meshwork of texts and communities. Thus literacies –and individual skills in orienting to Language – lie at the core of peer-review. The same goes for agonistic attitudes: science is based in emotional commitment. In exchange between parties, some structural relations must be dismissed, some ignored and some used to prompt constructive disagreement (e.g., Harnad, 1979). In reviewing, all parties can gain personal knowledge which, traditionally, can be addressed to the concerns of a sub-community or field that are orchestrated by an editor.

## Real Science Revisited

Science demands belief in scientific quality. Far from relying on neutral values or objective practices, attitudes have a crucial role in the making of knowledge that, for a community, is accorded objective validity. Arguably, this is why peer-review matters to science and, thus, the world’s knowledge sector. With such ideas in mind, I used mainstream literature to rethink the practice as a multi-scalar cognitive process. Although quality control matters, the function of peer review may be largely epistemic. Where the quality of science is under threat, other means are needed for quality control. Peer-review is, or should be, deeply concerned with the boundaries of scientific knowledge. As *more* than a normative process, I suggest that the recursive re-embodiment of review and writing rely on agonistic means of preserving scientific integrity. Crucially, one can investigate how symbolizations are aggregated in systems that link human agents, embodied activity and technology. In pilot work, I stress agonistic reviewing and, while sharing Ziman’s (2000) view that it is too early to evaluate post-academic science, I think that much can be gained from studying how, in fact, peer-review works. It is possible, for example, that some reviewers (and journals) focus on issues of knowledge/methods and others on the weighting given to favored social, economic and ethical outcomes. Indeed, this makes a parallel between science and agriculture so telling. By analogy, while some reviewers focus more quality science, others focus on what they think consumers want. For this reason, much post-academic science faces Alvesson’s (2013) problem of having nothing to say –especially if the goal as that of generating ‘evidence’ for predefined political, social and economic desiderata. Where servicing such a demand, peer-review is at odds with Ziman’s (2000) real science; growth of knowledge becomes more like cultivating coca leaves than managing the harvest of wheat or sorghum.

In stressing that peer-review is neither reliable, fair, nor a basis for predictive judgements. I say something that many will not want to hear. Even if this view *should* count as knowledge, power elites are likely to cling to Mertonian ideals. Pretending that peer-review is quality control suits many politicians, administrators, media and commercial interests. In a military image, peer-review is at the frontline of science. Further, while the topic is under-researched, many academics seem to support peer-review. Even if peer-culture is vanishing, its multi-scalar activities can be used to grasp what matters to a community, discipline or transdisciplinary field. What a sociologist calls ‘knowledge generation’ is, by hypothesis, amenable to redescription as the recursive aggregation of symbolizations that relevant players judge to be acceptable. From the systemic perspective, peer-review is epistemic. While enforcing conformity, the activity brings forth oversights, contradictions, and anomalies. Even if some parties focus on personal goals and financial rewards, peer-review serves bigger scientific needs. By maintaining agonistic ways of defending hard-earned knowledge, much can be achieved. On one hand, it can privilege data sets and ensembles of facts that drive the assumptions, practices and frames of a scientific field; on the other, the process can seed dissensus and its unpredictable outcomes. By hypothesis, peer-review is important in making scientific knowledge.

Science masks tensions between the known and the unknown: metaphors (e.g., input–output) and truisms (peer-review is a social process) hide ignorance. Much is gained from scrutiny of, not just knowledge claims, but of inconsistencies in argument, metaphors, rhetoric, and the sloppy use of wordings. But, this is not organized skepticism. In the resulting recursive events, affect has a major role: peer-review leads to collisions and chance events that set off what Whewell (1847) called the colligation of inference and Peirce (1982) normative extensions of perception. Peer-review prompts *abductive* processes that challenge opinions, knowledge claims, assumptions and metaphors. Bringing disorder to science, it permits non-conformity in those who care about science, content and their applications. Real science, and the best of its successors, is concerned as much with trust as it is in plugging research gaps. For Ziman (2000) a key motto is:“Be original”. Others, prefer to challenge the status quo. Provided that such pleasures are tempered by killjoy spirit, one can redefine problem spaces or, metaphorically, open up paths that can be used to ascend Mount Improbable. The normative power of peer-review can be rethought as linking embodiment and affect with social technologies and cultural products. On this view, symbolizations are crucial to all cultural ecosystems that depend on epistemic activity. A clearer view of peer-review may contribute to shaping events in the front line of the knowledge sector and, perhaps, resolving conflicts between Ziman’s (2000) real science and post-academic research whose primary concern is with meeting the needs of political, social and economic forces.

## Conflict of Interest Statement

The author declares that the research was conducted in the absence of any commercial or financial relationships that could be construed as a potential conflict of interest.

## Footnotes

1 As Giere (2004) argues, scientific knowledge (at least) is necessarily distributed between individuals and communities. Digitalization either reformats the digitized or transforms analog signals into digital formats (below, called ‘symbolizations’). As digitalized products lack semantic properties, they can neither be nor represent any kind of ‘knowledge’.

2 Even if the OED’s Sense 5 of science, is the … “dominant one in common use” (according to a 1987 Supplement), I view science as Wissenschaft and not around the usage first attested in 1867: “We shall … use the word science” in the sense that Englishmen so commonly give it; as expressing physical and experimental science, to the exclusion of the theological and metaphysical.” (W. G. Ward in The Dublin Review)

3 Ziman (2000, p. 2), the ‘Legend’ refers to a mythical ‘all conquering method’ that is exemplified by physics and chemistry. This narrow view obscures what most scientists do: for Ziman (2000), they use Merton’s (1942) ideals which, he argues, are being replaced by those of post-academic science: writing in 2000, he claims that it is too early to judge the effects of this change.

4 Cowley and Vallée-Tourangeau (2013) defend the usage by linking distributed cognition (Hutchins, 1995) to interactivity or sense-saturated coordination (see, Maturana’s (1978); Steffensen, 2013; Kirsh, 2015; to structural coupling (Maturana, 1978), one comes to focus on interactivity or sense-saturated coordination (Steffensen, 2013; Kirsh, 2015). In a cultural ecosystem (Hutchins, 2014), the impersonal aspect of language and experience makes cognition diachronic (Neumann and Cowley, 2013).

5 Linell’s (2004) *Written language bias in linguistics* is the classic work that identifies the widespread error. Emphasizing that language (and people) are fundamentally dialogical, he shows over 100 ways in which linguists are led astray when inscriptions are used as the basis for models of ‘language’.

6 On a distributed view, *wordings* are contrasted with *words*; whereas words are abstract, wordings are nonce events that are perceived and construed in the course of embodied and socio-cultural experience. While wordings are experiential, like words (and what are called ‘symbolizations’), they are *also* amenable to analysis as verbal patterns.

7 On May 25, 2015, this claim was verified on Wikipedia http://en.wikipedia.org/wiki/Peer_review

8 The Royal Society followed the footsteps of the *Le Journal des Savants*; however, this appeared less regularly than the *Transactions* and, so, its pioneering role can be questioned.

9 The remarkable blog http://retractionwatch.com/ offers reports of detected misdemeanors almost every day.

10 When ‘impact’ is defined epistemically, it is striking that, even in prestigious journals, many papers remain uncited (e.g., Hu and Wu, 2014); further, many citations merely echo a phrase, list similar findings or endorse a theory, writer or school (e.g., MacRoberts and MacRoberts, 2010). Pedagogically too, the impact of research wanes as it is separated from teaching.

11 In other terms, technological assessment standards (‘validation tests’) co-occur with the criteria that characterize Ziman’s (2000) ‘real science’ (evaluation tests). For example, well-presented statistical data may be elegant and significant and, yet, incompatible with desired social outcomes. For Pontille and Torny (2015), not only are tensions between validation and evaluation inevitable but, crucially, they influence reviewers. From a systemic perspective, therefore, choices of wordings, graphics and data at once ‘report’ (roughly, *validate*) and contribute to knowledge-making (*evaluation*).

12 This is the message: in spite of a huge literature – and many quantitative studies – the study of peer-review produces valid results that are *unclear*. While ineffective as quality control, peer-review drives much scientific quality. Accordingly, I reframe the practice as constraining cognition and, thus, part of knowledge-making.

13 Both referees dislike the example. One suggests that such a study can be challenged by testing ‘empirical validation or verification’. In a world of real-science, this might occur. However, it would be hard to fund such a study and, if one showed that full bladders had little effect on decision making, finding a publisher would be difficult. In my view, therefore, Shea’s (2011) voice deserves to be heard; accordingly, I leave it to the reader to make a judgment.

14 Calling it a ‘scientific plague’, the editors of *the Journal of Threatened Taxa* have recently proposed the development of a policy to prevent the citation of papers in known predatory journals (Raghavan et al., 2015).

15 When one invokes ‘external representation’, one focuses on forms and denotata (for discussion, see Kirsh, 2010). In contrast, symbolizations are perceived at an instant –for Peirce (1982), they are tokens that map onto types (e.g., sound-patterns, letters, graphics).

16 Dawkins (1997) uses this metaphor to describe his gene-centered view of natural selection; here I use it to describe how, in cultural time, communities settle on scientific content.

17 One read its hypothesis as “Symbolization influences later behavior and human life and peer-review is conceived as ‘Language’, which has normative influences over human beings”. The other referee claims to ‘find it difficult to summarize the author’s view which is both that peer-review is unfit for the purpose of quality assurance and, yet, “crucial to knowledge generation” and ‘assumed to guarantee good science’. The referee concluded that the ‘author could be said to set up one strawman to be replaced by another’.

18 These comments refer to much shorter draft of this paper –one that differs greatly from that presented here. Crucially, reviewers use an agonistic tone even when they are focused on neither content nor argument. This, I claim, is part of knowledge-making.

19 Reviewing also prompts the muting of my authorial voice: I reluctantly admit that even ‘weak’ (or computational) views of ‘embodiment’ views fit the hypothesis. In principle, people (or, if they exist, minds) might exploit the internal equivalents to ‘symbolizations’. Such a view would also have to explain how people/minds co-represent circumstantial aspects, ecosystemic connotations and, in some cases, covariant relations to practices and measures of objective validity.

20 Although I have cut the claim saying that ‘most new ideas are wrong’, this is not because I think it is false or important; rather, it is for reasons having to do with the overall coherence of the argument.
